# A Multiple Shape Memory Hydrogel Induced by Reversible Physical Interactions at Ambient Condition

**DOI:** 10.3390/polym9040138

**Published:** 2017-04-12

**Authors:** He Xiao, Chunxin Ma, Xiaoxia Le, Li Wang, Wei Lu, Patrick Theato, Tuoping Hu, Jiawei Zhang, Tao Chen

**Affiliations:** 1 Department of Chemistry, College of Science, North University of China, 030051 Taiyuan, China; xiaohe@nimte.ac.cn; 2 Key Laboratory of Marine Materials and Related Technologies, Ningbo Institute of Material Technology and Engineering, Chinese Academy of Sciences, 315201 Ningbo, China; machunxin@nimte.ac.cn (C.M.); lexiaoxia@nimte.ac.cn (X.L.); wangli@nimte.ac.cn (L.W.); luwei@nimte.ac.cn (W.L.); 3 Institute for Technical and Macromolecular Chemistry, University of Hamburg, Bundesstraße 45, 20146 Hamburg, Germany; theato@chemie.uni-hamburg.de

**Keywords:** multiple shape memory, chitosan, microcrystal, chain entanglement, hydrogel

## Abstract

A novel multiple shape memory hydrogel is fabricated based on two reversible physical interactions. The multiple shape memory property is endowed by a simple treatment of soaking in NaOH or NaCl solutions to form chitosan microcrystal or chain-entanglement crosslinks as temporary junctions.

## 1. Introduction

Shape memory polymers (SMPs) are a unique class of smart materials. They have the capability to recover to the initial shapes from temporary shapes triggered by external stimuli [1,2,3,4,5,6,7,8,9,10], so SMPs have attracted increasing attention in the fields of smart actuators, drug release, textiles, aerospace, etc. [11,12,13,14]. Most conventional SMPs are thermo-responsive polymers, in which vitrification or crystallization of switching domains are applied as temporary crosslinks to stabilize the deformed shapes, and the shape recovery is induced by heat [15,16,17,18,19,20,21]. As heat is not a convenient stimulus in practical biomedical and textiles applications, reversible interactions, such as metal-ligand coordination, host-guest interactions, and dynamic covalent bonds, have been applied to realize shape memory behavior in mild conditions [22,23,24,25,26,27,28,29,30,31]. Moreover, since the potential applications of SMPs are normally determined by the number of temporary shapes that can be fixed, new efforts have been devoted to developing SMPs with a multiple shape memory effect, whereby two or more temporary shapes can be stabilized. In our previous work, phenylboronic-diol ester bonds and the chelation of alginate with Ca^2+^, Schiff base bonds, and the coordination between chitosan and metal ions are respectively combined as two non-interfering reversible switches in one system, and a triple shape memory effect is thus achieved [30,31]. However, it is still challenging to design and fabricate more SMPs with two or more non-interfering reversible interactions. To avoid a complex molecular design and synthesis of new polymers, new strategies have to be developed to achieve a multiple shape memory effect at ambient condition.

**Scheme 1:**
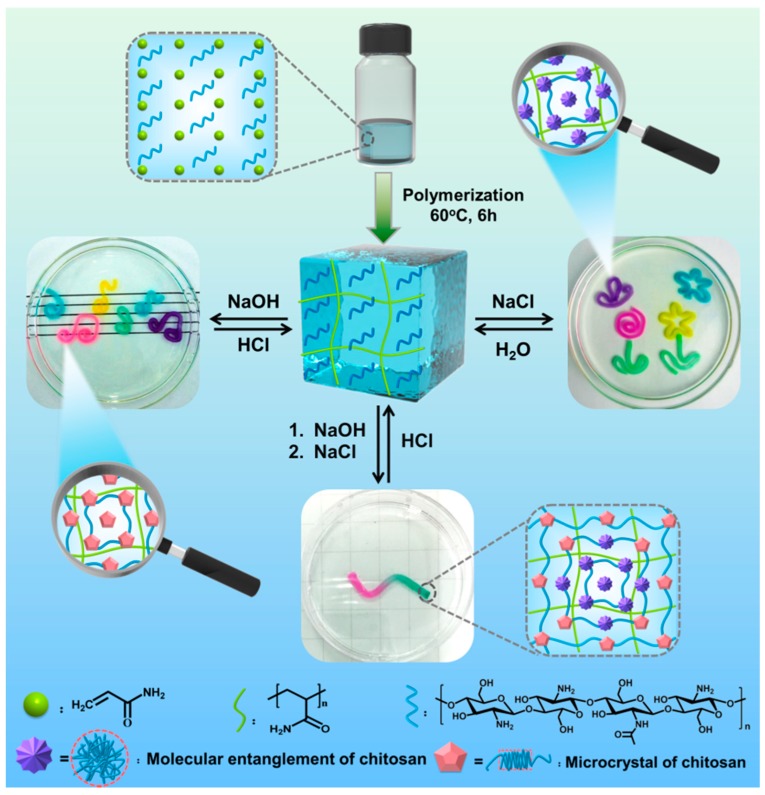
The mechanisms of the shape memory hydrogel based on two physical interactions. Acrylamide was polymerized in the presence of chitosan. The reversible CS microcrystalline crosslinks and CS chain-entanglement endowed the hydrogel with a programmable multiple shape memory ability.

Herein, we present a novel and universal soaking strategy to endow a hydrogel programmable multiple shape memory property. As depicted in Scheme 1, the developed hydrogel (PAAm-CS) was prepared by free radical polymerization of acrylamide (AAm) in the presence of biopolymer chitosan (CS). As chitosan is able to self-assemble into microcrystal and chain entanglement by simple soaking in alkaline and saline solutions [32,33], respectively, the two physical interactions were applied to stabilize temporary shapes and to endow the hydrogel shape memory ability. Moreover, taking advantage of the diffused transition mechanism, a multiple shape memory effect was successfully accomplished. In addition, by combining the two physical interactions, the programmable multiple shape memory effect was achieved. To the best of our knowledge, our system is the first to take a simple and universal soaking strategy to realize a multiple shape memory effect at ambient condition, which will broaden the list of SMPs and inspire the design and fabrication of novel versatile SMPs.

## 2. Materials and Methods

### 2.1. Materials

Chitosan (deacetylation: 80–95%, viscosity: 50–800 mPa s), acetic acid, acrylamide (AAm), hydrochloric acid (HCl), calcium chloride (CaCl_2_), potassium chloride (KCl), potassium nitrate (KNO_3_), and sodium chloride (NaCl) were obtained from Sinopharm Chemical Reagent Co. Ltd. (Shanghai, China). Ammonium, ammonium persulfate (APS), *N*,*N′*-Methylene *bis*(acrylamide) (*Bis*), sodium hydroxide, tert-butyl carbazate rhodamine B (red dye), methyl violet (purple dye), tartrazine (yellow dye), and erioglaucine disodium salt (blue dye) were purchased from Aladdin (Shanghai, China). APS and AAm were used after recrystallization, and the other chemicals were used without further purification.

### 2.2. Characterization

Scanning electronic microscopy (SEM) measurement was conducted with a Hitachi S4800 microscope. The rheological measurements were performed on a Haake MARSIII rheometer equipped with a geometry of 25 mm parallel plates at 25 ± 0.1 °C. The tensile and compression tests were conducted on a tensile-compressive tester (Instron 5567, Instron, Norwood, MA, USA). The samples for the tensile tests were prepared in a dumbbell shape 50 mm in length and 4 mm in width and were then measured at an extension rate of 50 mm/min until snapped. The samples for the compression tests were prepared in a cylinder shape with a length of 8–10 mm and a diameter of 10 mm and were measured at a compression rate of 10% original height/min and a final compressive strain is 98%.

### 2.3. Preparation of the Hydrogel

The chitosan (CS) solution (4%, *w*/*v*) was prepared by dissolving a certain amount of chitosan in acetic acid 2.1% (*w*/*v*) aqueous solution and stirred for about 5 h. Typical CS-AAm hydrogel was prepared by thoroughly mixing CS aqueous solution, AAm (600 mg), methylene-bis-acrylamide (30 μL, 10 mg/mL) as the crosslinker and APS (12 μL, 500 mg/mL) as the initiator with deionized water. The mixture was polymerized at 60 °C for 6 h.

### 2.4. The Shape Fixity Ratio

The quantitative shape memory property was determined according to the report method [23,34]. Shape fixity ratio (*R*_f_) and shape recovery ratio (*R*_r_) were defined by the following equation:

*R*_f_ = θ_t_/θ_d_ × 100%

*R*_r_ = (θ_d_ − θ_f_)/θ_d_ × 100%

where θ_d_ is the deformed angle, θ_t_ is the temporarily fixed angle, and θ_f_ is the final angle.

## 3. Result and Discussion

### 3.1. The Microscopic Changes and Mechanical Properties of PAAm-CS Hydrogel

**Figure 1:**
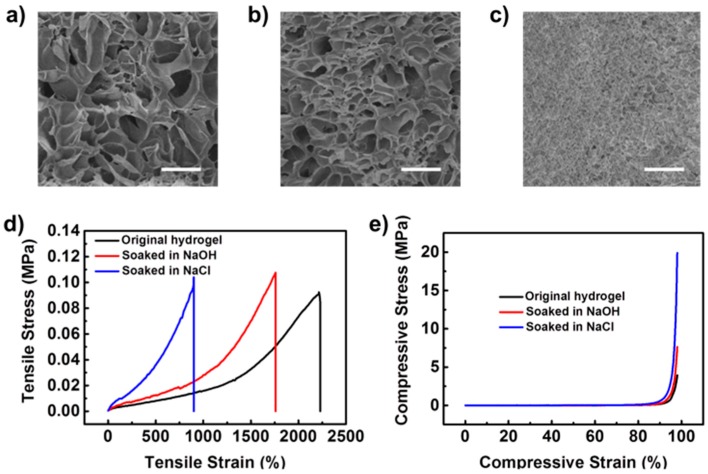
The cross-section SEM images of (**a**) original PAAm-CS hydrogel; (**b**) PAAm-CS hydrogel soaked in NaOH (0.75 mM); (**c**) PAAm-CS Hydrogel soaked in saturated NaCl. (Scale bars, 10 μm) (**d**) Tensile stress–strain curves and (**e**) compressive stress–strain curves of different hydrogels.

In order to confirm the formation of microcrystal and chain entanglement, scanning electron microscopy (SEM), tensile and compression test, and rheological measurements were performed. As shown in Figure 1, compared with the original PAAm-CS hydrogel (Figure 1a), the microstructure of hydrogel after soaking in NaOH (Figure 1b) or NaCl (Figure 1c) solution shows a higher density and smaller pores, which suggests the formation of a new second crosslinked network by microcrystal or chain entanglement, and the results are consistent with previous reports [31]. Moreover, the mechanical properties of the hydrogels are also changed with the introduction of the two physical interactions (Figure S1), the tensile strain shows a downtrend (Figure 1d), the compressive strength presents an uptrend (Figure 1e), and the chain entanglement seems to have more obvious influence to the tensile strain and the compressive strength. In addition, the formation of microcrystal or chain entanglement is also supported by rheological measurements (Figure S2).

### 3.2. Dual Shape Memory Effects of the PAAm-CS Hydrogel

**Figure 2:**
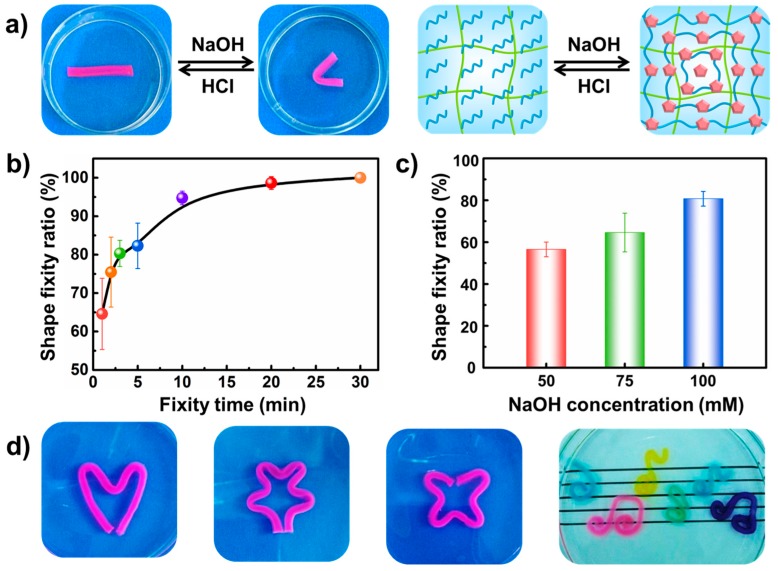
(**a**) The shape memory behavior and mechanism based on chitosan physical microcrystalline crosslink. (**b**) Variation of the shape fixity ratios of the PAAm-CS hydrogels as a function of the fixity time in NaOH solution (75 mM). (**c**) Variation of the shape fixity ratios as a function of concentration of NaOH solution with a shape fixity time of 1 min. (**d**) Images of the more complex temporary shapes of PAAm-CS hydrogel fixed by NaOH (75 mM).

Recently, it has been reported that the amine groups of chitosan will deprotonated in alkaline solution and induce the formation of physical microcrystalline crosslinks [32,33,35,36], the crystalline network junctions are expected to function as temporary crosslinks and endue the as-prepared PAAm-CS hydrogel shape memory capability. As schematically depicted in Figure 2a, a straight stripe sample was deformed into a “U” shape and immersed into NaOH solution (75 mM), the temporary shape can be fixed within 1 min due to the rapid formation of CS microcrystalline crosslinks. Once transferred into HCl solution (100 mM), the microcrystalline physical crosslink junctions will be destroyed and the hydrogel will recover to its original straight shape (Figure 2a and Figure S3). Since the shape memory effect of the PAAm-CS hydrogel is on the basis of the external base diffusion, two variable changes in the control of the shape fixity ratio can be expected. One variable change is the fixity time of the temporary shape. As shown in Figure 2b, the shape fixity ratio increases with increasing fixity time and reaches about 95% after immersing into NaOH solution (75 mM) for 10 min. If the shape fixity time is kept at 1 min, the shape fixity ratio increases with increasing concentration of NaOH solution (Figure 2c). The excellent shape memory effect of the hydrogel encourages us to explore the ability to fix the more complex shapes. Figure 2d perfectly shows that the straight samples dyed in pink were respectively deformed into various temporary shapes such as “heart”, “tree”, and “butterfly” under external stress and fixed by the CS microcrystalline physical crosslink junctions. When they were immersed into HCl solution, the complex temporary shapes were recovered to the original straight shapes (Figure S3). The shape recovery ratio increases with increasing immersing time and reached almost 100% within 12 min (Figure S3e). In addition, more complicated temporary shapes such as “music note” were also stabilized by the treatment of NaOH and recovered to the original straight shapes under the trigger of HCl.

**Figure 3:**
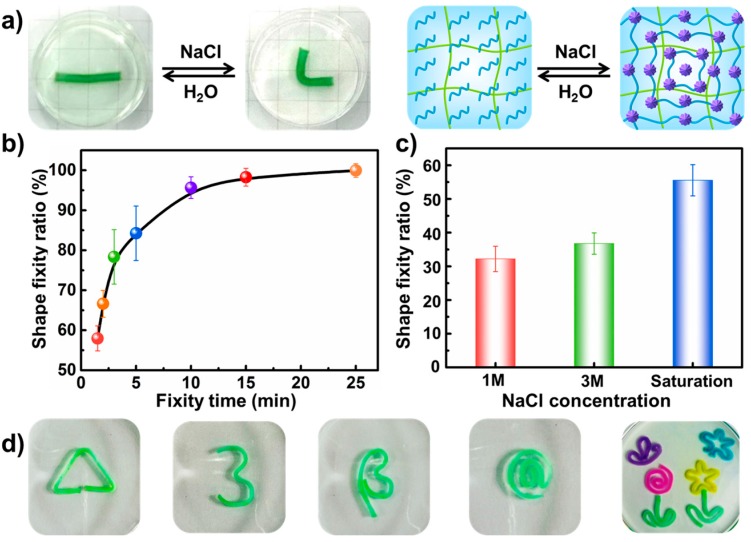
(**a**) The process and mechanism of the shape memory behavior with chitosan chain-entanglement as temporary crosslinks. (**b**) Variation of the shape fixity ratios of the PAAm-CS hydrogels as a function of the fixity time in saturated NaCl solution. (**c**) Variations of the shape fixity ratios as a function of concentration of NaCl solution with a shape fixity time of 1.5 min. (**d**) Images of the more complicated temporary shapes fixed by saturated NaCl solution.

Since CS chains in the PAAm-CS hydrogel can self-assemble to form physical entanglement crosslinks under the treatment of saturated saline solution [31], the chain entanglement physical networks is expected to undergo reversible arranging and disassociating process in the developed PAAm-CS hydrogel, endowing the hydrogel shape memory property. Figure 3a shows that a straight hydrogel sample was bent to the “U” shape and immersed into the saturated NaCl solution, the temporary shape was fixed within 90 s. Then, the sample was transferred into deionized water, the temporary shape recovered to its original shape after 20 min because of the diffusion of NaCl into water, and lead to the disassociation of the CS chain entanglement (Figure S4). Moreover, because the temporary physical crosslink junctions result from the diffusion of the saline solution into the hydrogel, the shape fixity ratio was controlled through changing the soaking time or the concentration of the NaCl solution. As shown in Figure 3b, extending the shape fixing time will allow more water in hydrogel to separate out, and the CS intermolecular aggregation that serves as junction points in hydrogel will increase and lead to the increment of the shape fixity ratio. With the concentration of the saline solution increasing, the separation of the water in hydrogel will also accelerate and the shape fixity ratio will increase as expected (Figure 3c). As a result, complex shapes such as “triangle”, “β”, and “@” were fixed (Figure 3d). It is worth noticing that the more complicated “butterfly loves flower” shape was also stabilized with a longer fixing time. Once expose to deionized water, all of these fixed complex shapes will recover to the original shapes (Figure S4). The shape recovery ratio increases as immersing time increases, and reaches almost 100% within 10 min (Figure S4e).

According to the formation mechanism of the CS physical entanglement crosslink, it is envisioned that immersion into saturated saline solutions is a universal method of endowing the PAAm-CS hydrogel with shape memory performance. To verify this proposal, straight stripes of hydrogels were deformed to a “U” shape and immersed in the saturated CaCl_2_, KCl, and KNO_3_ solutions, respectively; all temporary shapes were then fixed within 90 s (Figure S5a). Once transferred into deionize water, the temporary shape recovered to its original shape (Figure S6). Since the salting out effect relates to the ionic strength of saline solutions, the shape fixity capacity varies with different saline solutions (Figure S5b). CaCl_2_ solution with the highest saturation concentration represents the superior shape memory ability, while the KNO_3_ solution has the minimal capacity to fix the temporary shape because of the lowest ionic strength. Therefore, the PAAm-CS hydrogel was endowed with a multi-responsive shape memory effect under the stimulus of various saline solutions.

### 3.3. Multiple Shape Memory Effect of the PAAm-CS Hydrogel

**Figure 4:**
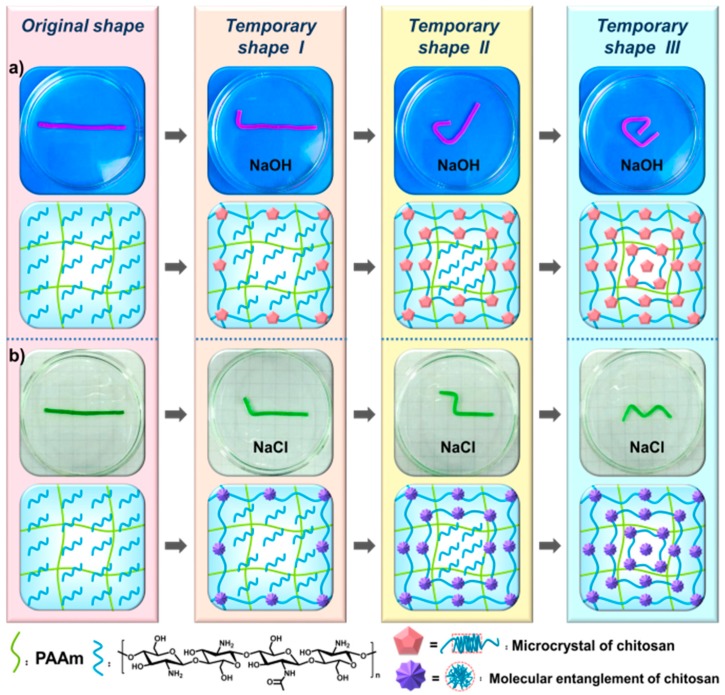
The shape memory processes and mechanisms of the multiple shape memory behavior. The multiple shape memory behavior based on (**a**) the CS physical microcrystalline crosslink and (**b**) the CS chain-entanglement.

In the shape memory process induced by both NaOH solution and saturated NaCl solution, the stimulus diffuses from the outside of the hydrogel to the inside, so it is expected that a multiple shape memory effect may be achieved by the diffused transition mechanism. In order to clearly confirm the expectation, the multiple shape memory ability of the PAAm-CS hydrogel was first explored by fixing the temporary shapes step by step upon the stimulus of NaOH solution. As shown in Figure 4a, a straight stripe of hydrogel dyed in pink is first deformed and immersed into NaOH solution for 1 min, and Temporary Shape I was fixed because of the formation of CS physical microcrystalline crosslinks on the surface of the hydrogel. The hydrogel stripe is bent again and transferred into NaOH solution for another 1 min to stabilize Temporary Shape II via CS physical microcrystalline crosslink interactions at the deeper levels of the hydrogel. Finally, the hydrogel stripe is deformed to Temporary Shape III and subsequently put into the NaOH solution once again, Temporary Shape III will be fixed because the deepest CS chains in the hydrogel self-assemble into microcrystalline structures. After being sequentially transferred into HCl solution, the hydrogel will gradually recover from Temporary Shape III to the original shape (Figure S7a). A similar multiple shape memory performance was also accomplished by applying CS chain entanglement as temporary crosslinks upon the stimulus of saturated NaCl solution (Figure 4b). After being sequentially transferred into the deionized water, the hydrogel stripe will gradually recover to the original straight shape because of the dissociation of CS chain-entanglement physical crosslink (Figure S7b). Moreover, the multiple shape memory performance can be repeated at least 3 times without any distinct degradation (Figures S8 and S9). These results vividly present the multiple shape memory properties of the PAAm-CS hydrogel based on the two physical interactions, suggesting the potentially versatile applications.

### 3.4. Triple Shape Memory Effect of the PAAm-CS Hydrogel

**Figure 5:**
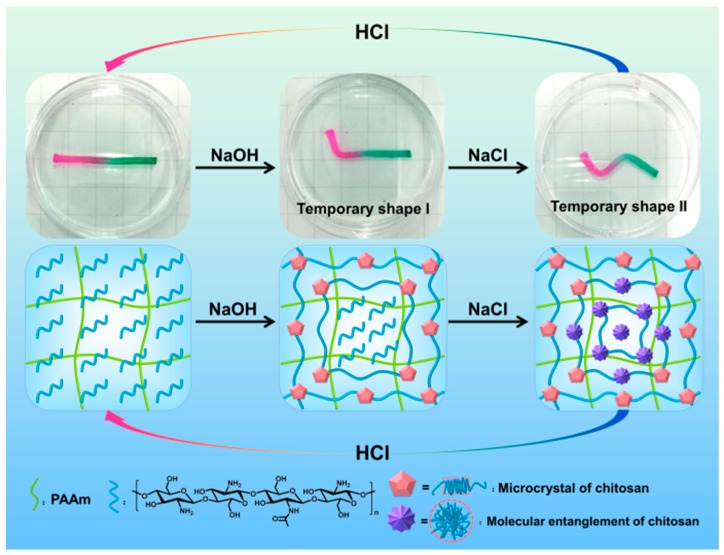
The process and mechanism of the programmable triple shape memory and recovery.

Besides the excellent shape memory performance achieved by each physical interaction, programmable triple shape memory of the PAAm-CS hydrogel can also be realized by combining the two physical interactions. As shown in Figure 5, a stripe-shaped hydrogel dyed in pink and green is immersed in the NaOH solution with 1 min to fix Temporary Shape I due to the formation of CS microcrystalline physical crosslink, the hydrogel is then deformed to Temporary Shape II and subsequently transferred into the saturated NaCl solution, and Temporary Shape II will be fixed after 90 s via the CS physical chain-entanglement crosslink. The complex “N” temporary shape directly recovered to the original straight stripe when transferring into the HCl solution (Figure 5 and Figure S10). This is because the external acid stimulus is not only a neutralization factor to alkaline but also a good diluent agent to NaCl; as a result, the two reversible physical interactions can be simultaneously destroyed. Moreover, the triple shape memory behavior can be repeated at least 3 times without any distinct degradation (Figure S11).

## 4. Conclusions

In summary, a novel hydrogel with multiple shape memory effect was successfully prepared by applying a universal soaking strategy. The hydrogel was fabricated based on two reversible physical interactions, the CS microcrystal, and chain-entanglement interactions, which acted as temporary physical crosslinks to stabilize the deformed shape of the hydrogel and to render the hydrogel with dual shape memory behaviors. Moreover, taking advantage of the diffuse transition mechanism, multiple shape memory effect was also accomplished via a sequent treatment of the hydrogel using NaOH or NaCl solutions. In addition, a programmable triple shape memory performance was successfully achieved by combining the two physical interactions. Taking advantage of the two reversible physical interactions of chitosan, we offered a novel and simple strategy to achieve multiple shape memory effect at ambient condition, thereby expanding the potential applications of SMPs.

## Supplementary Materials

The following are available online at www.mdpi.com/2073-4360/9/4/138/s1. Figure S1: The mechanical properties of different hydrogels; (a–c) Photographs of finger compression; (d–f) photographs of stretched hydrogels. Figure S2: Storage modulus (G′) and loss modulus (G′′) of original PAAm-CS hydrogel and hydrogels soaked in NaOH or NaCl solution, respectively. Figure S3: (a–d) The shape recovery procedures of PAAm-CS hydrogels with different temporary shapes in 100 mM HCl; (e) The shape recovery ratio as a function of time in 100 mM HCl. Figure S4: (a–d) The shape recovery procedures of PAAm-CS hydrogels with different temporary shapes in deionized water. (e) The shape recovery ratio as a function of time in deionized water. Figure S5: (a) The shape memory behavior based on the CS chain-entanglement induced by various saturated saline solutions. All the dashed lines indicate the temporary shape hydrogel will recover in the deionized water; (b) The shape fixity ratios based on the CS chain-entanglement as a function of saturated saline solutions. Figure S6: The recovery processes of the PAAm-CS hydrogel in deionized water, in which the temporary shapes are fixed by different saturated salt solutions. Figure S7: The recovery procedures of the PAAm-CS hydrogel with different stimuli; (a) The temporary shape was fixed by CS physical microcrystal crosslink interaction and the shape recovery is induced by HCl (100 mM); (b) The temporary shape was fixed by CS chain-entanglement crosslink interaction and the shape recovery is carried out in deionized water; Figure S8: Cycled multiple shape memory behavior of the PAAm-CS hydrogel via CS physical microcrystal crosslink interaction. NaOH solution (75 mM) and HCl (100 mM) were used for shape memory and shape recovery procedures, respectively. Figure S9: Cycled multiple shape memory behavior of the PAAm-CS hydrogel via CS chain-entanglement crosslink interaction. Saturated NaCl solution and deionized water were used for shape memory and shape recovery procedures, respectively. Figure S10: The shape recovery procedures of the triple-shape memory hydrogel in HCl solution (100 mM). Figure S11: Cycled triple shape memory behavior of the PAAm-CS hydrogel. The straight stripe of hydrogel was dyed in pink and green at the ends. The pink end of the temporary shape was fixed by NaOH solution (75 mM) with 1 min, and the green end was fixed by saturated NaCl within 2 min. When the hydrogel stripe was immersed in the HCl solution (100 mM), the temporary shape recovered to its original shape.
