# Supplementary material for: A Multiple Shape Memory Hydrogel Induced by Reversible Physical Interactions at Ambient Condition
Source: Polymers (Basel). 2017 Apr 12;9(4):138. doi: 10.3390/polym9040138 (PMC6432359; doi:10.3390/polym9040138)
Supplement: Supplementary file 1 [file polymers-09-00138-s001.pdf]

Supplementary Materials: A Multiple Shape Memory Hydrogel Induced by Reversible Physical Interactions at Ambient Condition

He Xiao, Chunxin Ma, Xiaoxia Le, Li Wang, Wei Lu, Patrick Theato, Tuoping Hu, Jiawei Zhang and Tao Chen

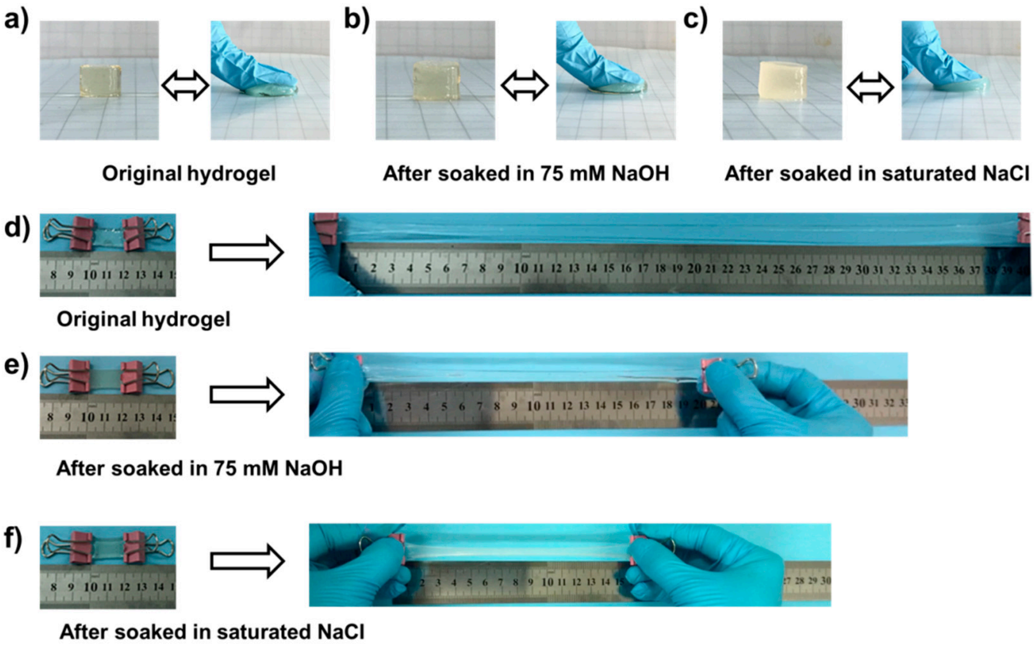

Figure S1. The mechanical properties of different hydrogels. (**a**)–(**c**) Photographs of finger compression; (**d**)–(**f**) Photographs of stretched hydrogels.

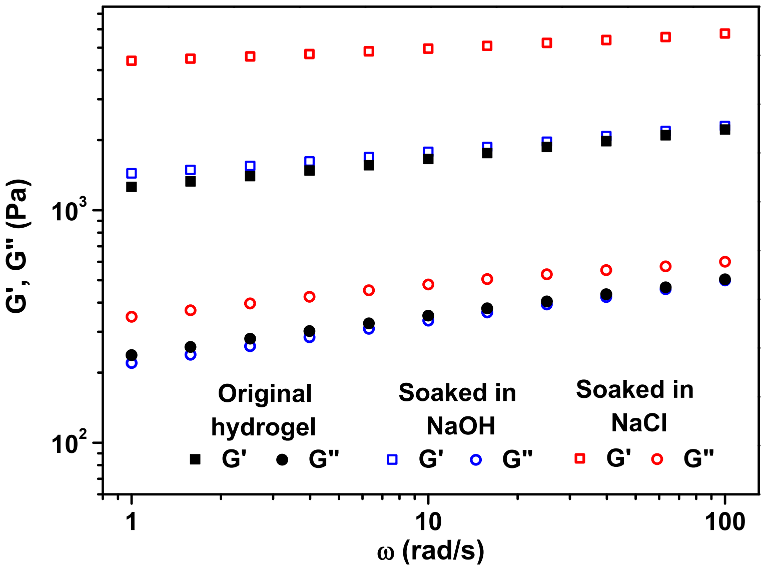

Figure S2. Storage modulus (G') and loss modulus (G'') of original PAAm-CS hydrogel and hydrogels soaked in NaOH or NaCl solution, respectively.

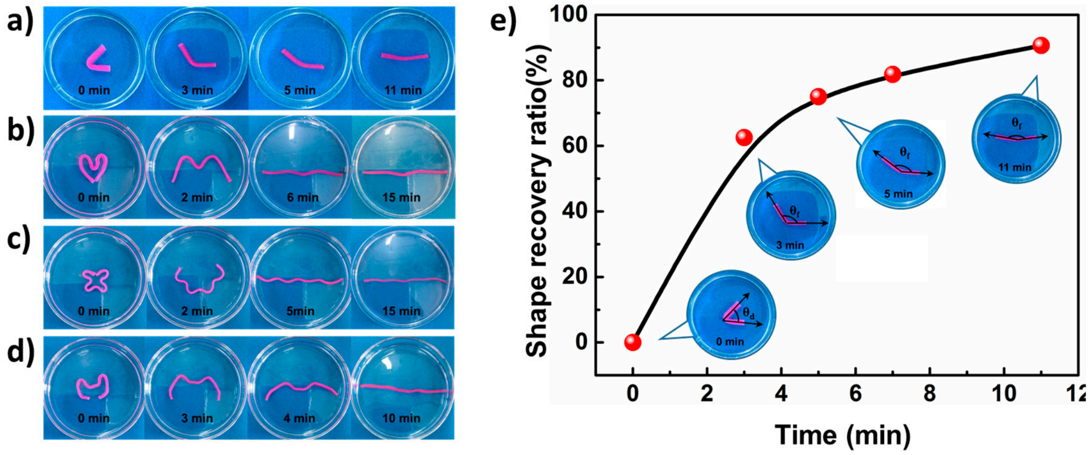

Figure S3. (**a**)–(**d**) The shape recovery procedures of PAAm-CS hydrogels with different temporary shapes in 100 mM HCl; (**e**) The shape recovery ratio as a function of time in 100 mM HCl.

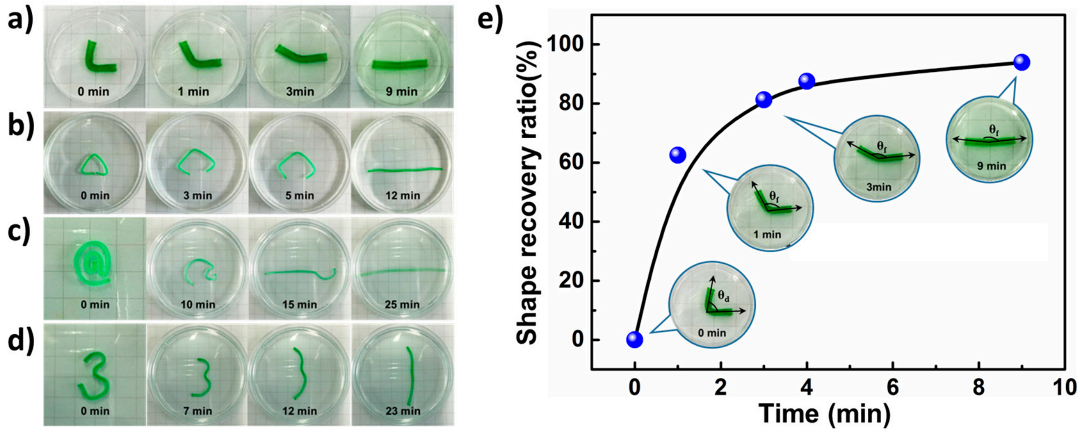

Figure S4. (**a**)–(**d**) The shape recovery procedures of PAAm-CS hydrogels with different temporary shapes in deionized water; (**e**) The shape recovery ratio as a function of time in deionized water.

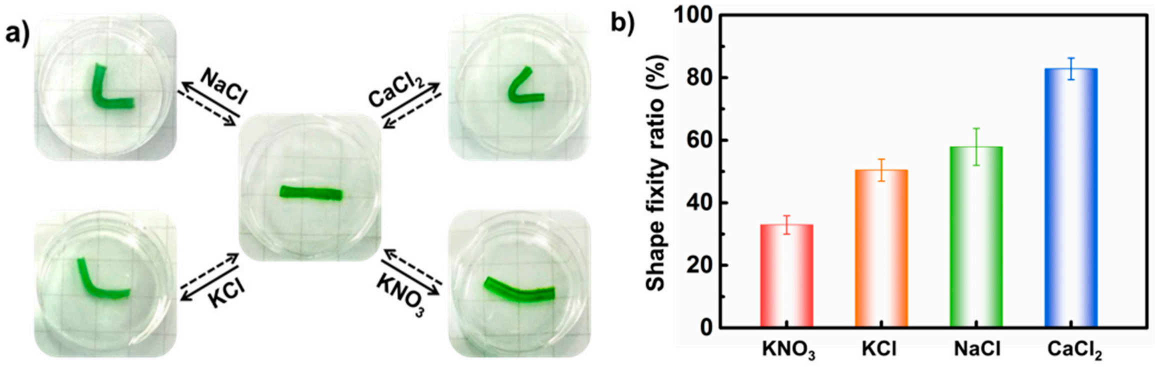

Figure S5. (**a**) The shape memory behavior based on the CS chain-entanglement induced by various saturated saline solutions. All the dashed lines indicate the temporary shape hydrogel will be recovered in the deionized water; (**b**) The shape fixity ratios based on the CS chain-entanglement as a function of saturated saline solutions.

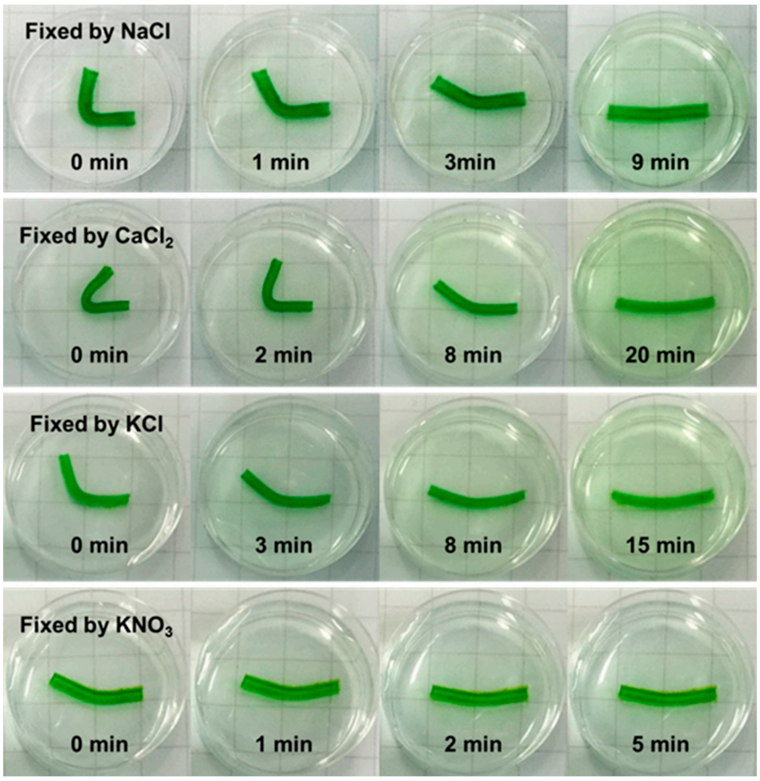

Figure S6. The recovery processes of the PAAm-CS hydrogel in deionized water, in which the temporary shapes are fixed by different saturated salt solutions.

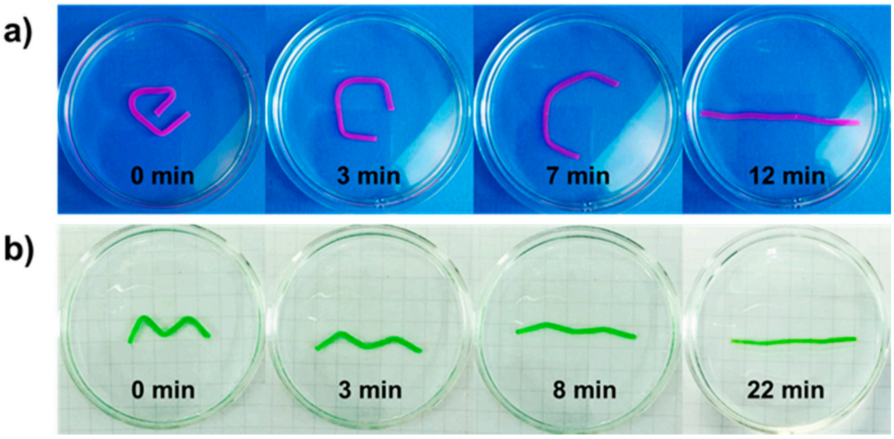

Figure S7. The recovery procedures of the PAAm-CS hydrogel with different stimuli. (**a**) The temporary shape was fixed by CS physical microcrystal crosslink interaction and the shape recovery is induced by HCl (100 mM); (**b**) The temporary shape was fixed by CS chain-entanglement crosslink interaction and the shape recovery is carried out in deionized water.

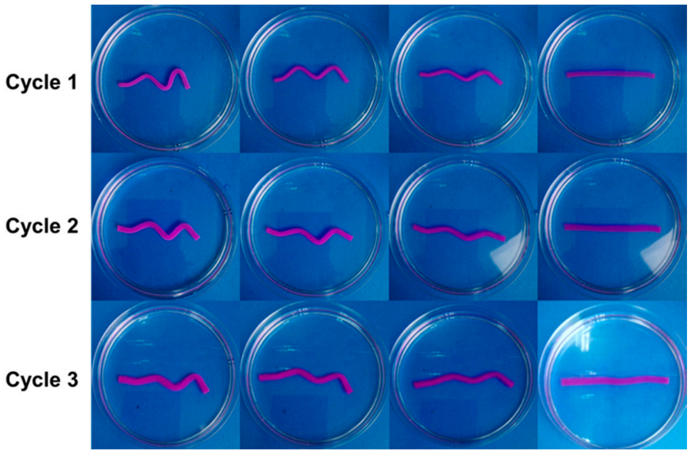

Figure S8. Cycled multiple shape memory behavior of the PAAm-CS hydrogel via CS physical microcrystal crosslink interaction. NaOH solution (75 mM) and HCl (100 mM) were used for shape memory and shape recovery procedures, respectively.

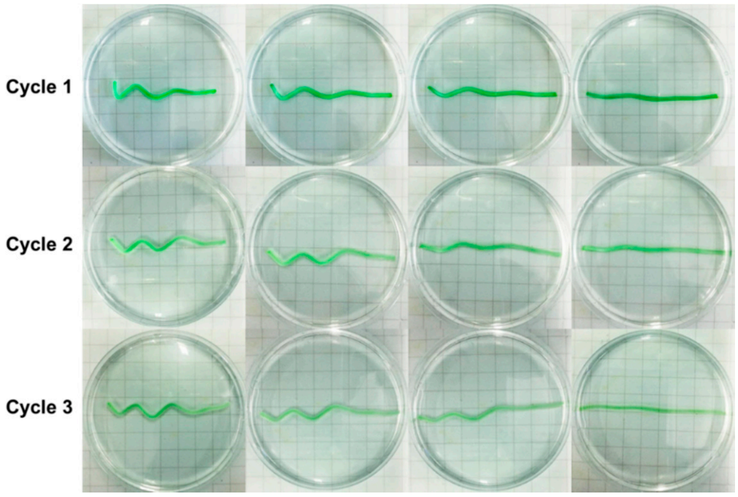

Figure S9. Cycled multiple shape memory behavior of the PAAm-CS hydrogel via CS chain-entanglement crosslink interaction. Saturated NaCl solution and deionized water were used for shape memory and shape recovery procedures, respectively.

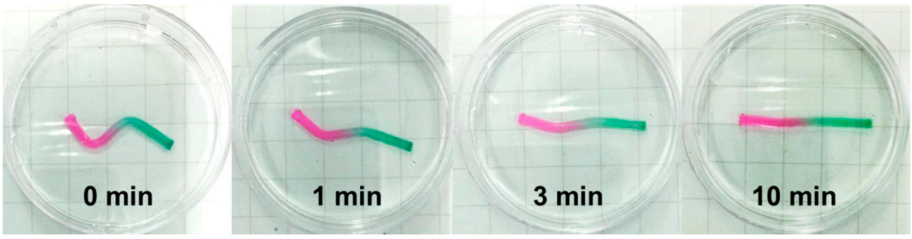

Figure S10. The shape recovery procedures of the triple-shape memory hydrogel in HCl solution (100 mM).

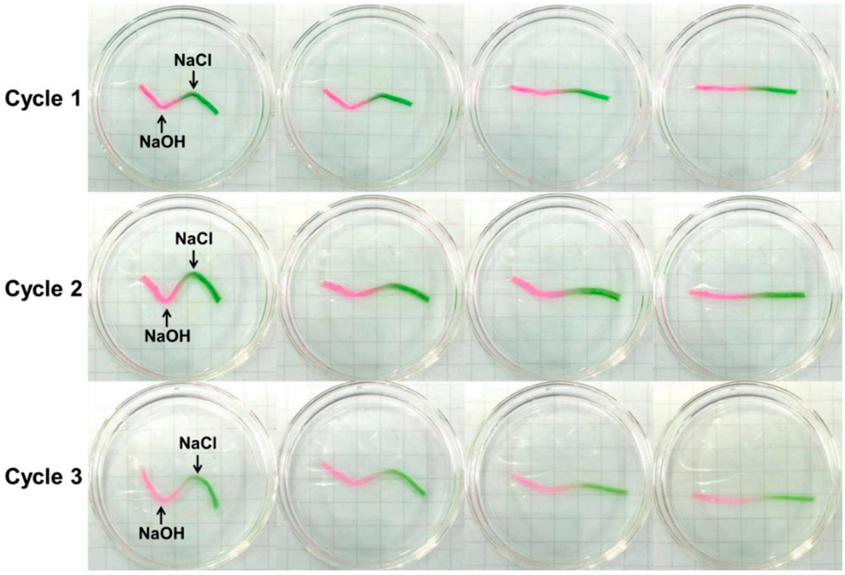

Firgure S11. Cycled triple shape memory behavior of the PAAm-CS hydrogel. The straight stripe of hydrogel was dyed in pink and green at the ends. The pink end of the temporary shape was fixed by NaOH solution (75 mM) with 1min and the green end was fixed by saturated NaCl within 2 min. When the hydrogel stripe was immersed in the HCl solution (100 mM), the temporary shape could be recovered to its original shape.
